# Effect of aqueous and organic solvent extraction on *in-vitro* antimicrobial activity of two varieties of fresh ginger (*Zingiber officinale*) and garlic (*Allium sativum*)

**DOI:** 10.1016/j.heliyon.2022.e10457

**Published:** 2022-08-28

**Authors:** Jolly Oder Akullo, Beatrice Kiage, Dorothy Nakimbugwe, John Kinyuru

**Affiliations:** a Department of Animal Production and Management, Faculty of Agriculture and Animal Sciences, Busitema University, PO box 203, Soroti, Uganda; b Department of Human Nutrition Sciences, School of Food and Nutrition Sciences, Jomo Kenyatta University of Agriculture and Technology, P.O. Box 62 000, 00200 Nairobi, Kenya; c Department of Food Technology and Nutrition, School of Food Technology, Nutrition and Bio-Engineering, Makerere University, PO Box 7062, Kampala, Uganda; d Department of Food Science and Technology, School of Food and Nutrition Sciences, Jomo Kenyatta University of Agriculture and Technology, P.O. Box 62 000, 00200 Nairobi, Kenya

**Keywords:** Antimicrobial activity, Minimum inhibitory concentration, Agar well diffusion method, Pathogens

## Abstract

The current state of antimicrobial resistance to synthetic antimicrobial drugs has led to renewed interest in natural antimicrobial compounds. Antimicrobial activity of extracts of (local and hybrid) ginger and garlic was investigated using the agar well diffusion method against *Staphylococcus aureus, Escherichia coli* and *Candida albicans.* Aqueous and organic solvent extracts of both varieties of ginger and garlic exhibited varied and concentration-dependant antimicrobial activity. Inhibition zones at 25 mg/mL varied significantly against the microorganisms, being highest on *C. albicans*; 18.00 ± 2.00 to 30.67 ± 1.16 mm for acetone extracts and raw juice of hybrid ginger and 19.67 ± 1.16 to 30.33 ± 1.53 mm for methanol and raw extracts of local garlic respectively. Minimum Inhibitory Concentration ranged from 2.5 to 10 mg/mL in garlic extracts. The study concluded that both varieties of ginger and garlic possess antimicrobial substances, though ginger is more potent as antifungal agent.

## Highlights

• Extracts exhibited varied concentration-dependant antimicrobial activity.
• Inhibition was higher for garlic compared to ginger extracts against bacteria.
• Activity of garlic ethanolic extracts compared favourably with garlic raw juices
• Minimum Inhibitory Concentration ranged from 2.5 to 10 mg/mL in garlic extracts

## 1 Introduction

Antimicrobials substances from plant origin are secondary metabolites that are produced and used by plants for protection. These secondary metabolites have various uses in medicine and food application (Tiwari and Rana, 2015). Plant antimicrobial substances have attracted a lot of attention from scientists as ingredients in human drugs (De Zoysa et al., 2019; Maharjan et al., 2012); and of great interest is the control of spoilage and pathogenic microorganisms that cause food borne infections and intoxications (Sofia et al., 2007; Thongson et al., 2005). This is particularly important due to the current state of antimicrobial resistance encountered in the food chain which poses a big risk to public health (Bennani et al., 2020; Oniciuc et al., 2019).

Traditionally, herbs and spices have been used in many communities for management of several aliments, including as antimicrobial substances (Dini, 2018; Dog, 2006; Leja and Czaczyk, 2016). When compared to the conventional antibiotics, herbs and spices are generally regarded as safe for humans; owing to their long term history of use in food preparation. Several herbs and spices are reported to be effective antimicrobials. For instance, studies have showed that cinnamon stick, oregano, clove, pomegranate peel and grape seed extracts exhibited antibacterial activity against foodborne pathogens with the Gram-positive bacteria being more sensitive than Gram-negative bacteria. *S. aureus* was reported as the most sensitive, while *E. coli* was the most resistant (Shan et al., 2007, 2009). The antibacterial activity of the extracts was closely associated with their phenolic content.

Ginger (*Zingiber officinale*) is a member of the family *Zingiberaceae*. It is widely distributed across the tropics of Asia, Africa, America and Australia where it is used as a spice and medicinal plant (Eun Jung and John, 2002). Studies on the diversity of ginger have shown that cultivated ginger exhibits variations in rhizomes and vegetative character and there is a large influence of environmental factors on the key bioactive compounds (Kizhakkayil and Sasikumar, 2011). On the other hand, garlic (*Allium Sativum*) is a bulbous perennial herb belonging to the family *Alliaceae.* Garlic is basically of two broad types; hard neck and the soft neck (Kshirsagar et al., 2018).

Hard neck garlics are characterized by hard, woody central stalks that extend down to the basal plate at the bottom of the bulb. Soft necked garlic have a non-woody pseudostem formed from overlapping leaf sheaths and rarely send up a flower stalk, unless when it is stressed by environmental conditions. Soft neck garlics are thought to have developed from the hard necked garlic. Detailed classification of the hard neck, soft neck and genetic variation of garlic is presented (Kshirsagar et al., 2018).

In Uganda, two varieties of ginger and garlic are available in the food markets and are commonly used as medicinal plants and condiments in food preparation to improve the flavor and aroma of food as well as the keeping quality (Muhanji, 2009; Safiriyu et al., 2018). The indigenous people (suppliers and consumers) refer to the varieties as “local” ginger and “hybrid” ginger, and “local” garlic and “hybrid/Chinese” garlic. The “local” ginger is used to describe the variety with small size rhizomes, thick brown skin, and yellow flesh/powder with a strong pungent aroma. “Hybrid” ginger describes the variety with big rhizomes, light yellow skin, off white flesh/powder and less pungent aroma. The “local” garlic refers to a locally grown cultivar; with small bulbs, purple colour, woody stalks and grown mostly in the colder parts of the country. The “hybrid” garlic is known by suppliers as “Chinese garlic.” The bulbs are big in size with silver like bulb scales, white in color and soft in nature. Hybrid variety is said to be imported into the country from China and has a good storage quality. The local and hybrid garlic varieties align with the classification of the hard neck and soft neck respectively as described by (Kshirsagar et al., 2018). Soft neck garlics have longer keeping quality due to a high number of protective shells, when compared to the hard neck garlic (Akan, 2019).

Several studies have reported on the antimicrobial activity of ginger and garlic (Akintobi et al., 2013; Indu et al., 2006; Khashan, 2014; Mohammed et al., 2019). There is wide spectrum of antibacterial activity of ginger rhizome against a range of Gram negative and Gram positive bacteria. However, conflicting reports exist about the antibacterial effectiveness of ginger against bacteria from different resources (Abdalla and Abdallah, 2018). Antifungal and antiviral activity of ginger is also reported (Gebreyohannes and Gebreyohannes, 2013). Zingiberene in ginger rhizome oils are the most active antibacterial component (El-baky et al., 2010); and the therapeutic effectiveness of garlic is attributed to its oil content and water soluble organosulfur compounds (Prati et al., 2014).

It is demonstrated that the antimicrobial effect of ginger and garlic are affected by extraction solvent, and method and concentration of bioactive compounds respectively (Ali Hasan, 2012; Bakht et al., 2011). It was reported that Chinese garlic demonstrated high potential as ingredients for food application due to high allicin content (Sommano et al., 2016). It is also confirmed that efficacy of plant product is greatly affected by the method of extraction, antimicrobial assay conditions, genetic variations, bacterial strains as well as its sources. Additionally, plants are affected by geographic variations, environmental conditions and physiological factors which influence theirs bioactive phytochemical compounds (Abdalla and Abdallah, 2018).

There is limited scientific evidence on the antimicrobial activity of the varieties of ginger and garlic used in Uganda, and this may affect its utilization and wide spread industrial application. Additionally, the ability of the different solvents to extract the active ingredients and their influence on the antimicrobial activity of these spices is reported to vary with the nature of the solvent (Bakht et al., 2011).

Therefore, this study aimed to evaluate the effect of extraction solvents on the antimicrobial activity of two varieties of fresh garlic and ginger locally available in Ugandan markets against common foodborne pathogens (*S. aureus, E. coli* and *C. albicans*).

## 2 Materials and methods

### 2.1 Plant material collection, pre-processing and extraction

Two varieties (local and hybrid) of fresh garlic cloves and ginger rhizomes were separately purchased from Lira City market (2.2581° N, 32.8874° E), in Northern Uganda. The market was sub divided into five (5) zones and from each zone, 0.5 kg of the local and hybrid variety of fresh ginger and garlic was purchased; hence a total of 2.5 kg of each variety was procured. Samples were packed in cool boxes in the 0.5 kg packages and transported to the Food biochemistry laboratory at Jomo Kenyatta University of Agriculture and Technology (JKUAT), Kenya. The respective samples were sorted to remove bad quality, soaked in tap water for 30 min, washed and rinsed under running water.

The skin of ginger and garlic samples was peeled prior to crushing/grating for extraction of phytochemicals. Extraction of ginger and garlic varieties was done separately using acetone, ethanol, methanol and water following standard procedures (Kang, 2015); with slight modifications. Briefly, 25 g of the respective crushed spice/variety was put in conical flasks and 100 ml of either of the extraction solvent (acetone, ethanol and methanol) added. Aqueous extracts were also made by mixing 25 g of the respective spice in 100 mL of distilled water. The flask containing solvent/aqueous-spice mixture was shaken at 300 rpm on a mechanical shaker (Ks 250 basic, Ika Labortechnik-orbital shaker, Japan) in the dark for 24 h; and thereafter the solution was filtered using whatman filter paper No 1. The filtrate of organic solvent was concentrated by rotary evaporation at 40 °C and refrigerated at 4 ± 1 °C in air tight bottles for subsequent analysis.

### 2.2 Microbial culture and media

Three food borne microorganisms; *S. aureus*, *E. coli* and *C. albicans* were used to check the antimicrobial activity of the spice extracts in this study. The microorganisms represented Gram-positive bacteria, Gram-negative bacteria and yeasts respectively. Standard microbial cultures were obtained from the Food Microbiology Laboratory, JKUAT; while media used in this study was from the Oxoid Limited, England.

The microbial solution from the mother culture was streaked using a sterile loop onto selective media plates to ensure growth of pure colonies. *Escherichia coli* was streaked on Mac-Ckonkey agar, *S. aureus* and *C. albicans* were streaked on nutrient agar. The agar plates were left to dry for about 3–5 min, inverted and incubated overnight at 37 °C for 24 h. Subsequently, a single pure colony was transferred from the streaked plates, into separate test tubes containing 10 mL of nutrient broth. The test tubes were then incubated at 37 °C for 24 h in order to boost growth of the respective microorganisms. The concentration of the actively growing broth cultures were adjusted by comparing with 0.5 McFarland standards using standard procedure to obtain a working culture of 10^6^ cfu/mL (Adetunde et al., 2014; Bakht et al., 2011). On the other hand, the process of culturing was repeated weekly during the study period in order to obtain viable working cultures.

### 2.3 Evaluation of the antimicrobial activity against bacteria and yeast

The agar well diffusion method was used to investigate the antimicrobial activity of the crude extracts according to the procedures described (Schumacher et al., 2018). Nutrient agar (NA) was used for the bacteria and Sabouraud Dextrose Agar (SDA) was used for the yeast. Twenty (20 mL) of the sterile agar media was transferred to the respective sterile petri-dishes and allowed to solidify. Inoculums (100 μL) of each microbial suspension was dispensed and evenly spread on the surface of the media in the agar plates. Using a sterile cork borer, four 9 mm wells were cut into the inoculated media in each agar plate; and the bottom of the well was sealed with molten nutrient agar.

#### 2.3.1 Inoculation with plant extracts

In the inoculated plates, 100 μL each of a specific spice extracts (25 mg/mL) was dispensed into two wells (treatment); and the same quantity of extract solvent was dispensed in the remaining two wells (negative control). This process was repeated for all the aqueous and organic solvents of the two varieties on the three test microorganisms in duplicates. Plates were properly labeled to show the media, microorganism inoculated, and spice extract dispensed into the wells; and left to dry/solidify for 1 h on the clean bench. Plates were then inverted and incubated at 37 °C for 24 h for bacterial assays and 25 °C for 72 h for yeast assay. After the incubation period, observed zones of inhibition around the well were measured using a Vernier caliper in mm, and this is the diameter of the growth free zones around the well, which is reported as the antimicrobial activity.

#### 2.3.2 Determination of the minimum inhibitory concentration (MIC) of the effective spice extracts

The plant extract that exhibited the strongest antimicrobial activity during screening stage at 25 mg/mL against all the test microorganisms was chosen for the determination of MIC using the agar well diffusion method. Different concentrations (15, 10, 5 and 2.5 mg/mL) of spice extracts were prepared separately using ethanol, methanol, acetone and water using the technique of microdilution as described by Lallemand et al. (2016). Sterile agar plates containing NA were also prepared and inoculated with 100 μL of *S. aureus* and *E. coli* culture separately, while SDA plates were used for *C. albicans*. Specific concentrations of the different extracts were added in each well as previously descried and the plates inverted and incubated aerobically. Antimicrobial activity of the different extracts was again assayed by measuring the zone of inhibition in mm for each concentration.

### 2.4 Data analysis

All measurements were done in triplicates for each microorganism. Values were reported as Mean ± Standard Deviation (SD). ANOVA tests were performed using GenStat software to determine significant differences in extracts. Statistical comparisons were separated using Duncan’s multiple range tests with Least Significant Differences (LSD) considered at *P ≤ 0.05***.**

## 3 Results

### 3.1 Antimicrobial activity of ginger and garlic in different solvent extracts

The antimicrobial activity of the two varieties of ginger rhizomes and garlic cloves extracted using different solvents are shown in Table 1. The study indicates that all the extracts from the local and hybrid ginger exhibited antimicrobial activity against tested microorganisms. Susceptibility of the three organisms (*S. aureus*, *E. coli* and *C. albicans*) to the solvent extracts differed significantly (*P* ˂ *0.05*).

**Table 1:** Antimicrobial inhibition activity of different solvent extracts (25 mg/mL) of ginger against different microorganisms.

Spice	Inhibition Zones (mm)			
	Solvent	*S. aureus*	*E. coli*	*C. albicans*
Hybrid ginger	Raw	20.00 ± 2.00^c^	13.33 ± 1.15^b^	30.67 ± 1.16^d^
	Acetone	16.00 ± 1.00^b^	11.33 ± 0.58^a^	18.00 ± 2.00^b^
	Ethanol	12.00 ± 1.00^a^	13.00 ± 1.00^ab^	20.00 ± 2.00^bc^
	Methanol	15.67 ± 2.08^b^	13.00 ± 1.00^ab^	19.33 ± 1.53^bc^
	Water	12.33 ± 1.52^a^	12.00 ± 1.00^ab^	14.67 ± 1.53^a^
Local ginger	Raw	11.67 ± 0.58^a^	11.67 ± 0.58^ab^	29.67 ± 0.58^d^
	Acetone	13.00 ± 1.00^a^	11.67 ± 0.58^ab^	21.67 ± 1.53^c^
	Ethanol	13.67 ± 2.5 2^ab^	12.00 ± 1.00^ab^	21.00 ± 2.65^bc^
	Methanol	12.00 ± 1.00^a^	12.67 ± 1.52^ab^	20.67 ± 1.56^bc^
	Water	11.67 ± 0.58^a^	12.00 ± 1.00^ab^	30.33 ± 0.58^d^
***P value***		***< 0.001***	***< 0.001***	***< 0.001***
Hybrid Garlic	Raw	32.67 ± 0.58^e^	33.00 ± 1.00^e^	26.33 ± 0.58^bcd^
	Acetone	25.33 ± 1.15^c^	25.67 ± 0.58^c^	26.00 ± 1.73^bcd^
	Ethanol	26.33 ± 2.08^c^	24.33 ± 1.16^c^	25.00 ± 1.00^b^
	Methanol	22.33 ± 0.58^b^	21.33 ± 1.16^c^	27.00 ± 1.00^cd^
	Water	21.33 ± 1.15^ab^	25.00 ± 1.73^d^	27.67 ± 0.58^d^
Local Garlic	Raw	29.67 ± 1.52^d^	29.67 ± 0.58^d^	30.33 ± 1.53^e^
	Acetone	20.67 ± 1.15^ab^	19.33 ± 2.31^ab^	20.67 ± 0.58^a^
	Ethanol	20.33 ± 2.30^ab^	20.33 ± 1.53^ab^	27.67 ± 0.58^d^
	Methanol	19.67 ± 1.52^a^	18.33 ± 1.53^a^	19.67 ± 1.16^a^
	Water	20.33 ± 0.58^ab^	24.00 ± 1.00^c^	25.33 ± 0.58^bc^
***P value***		***<.0.019***	***<.0.038***	***<.0.001***

Values = Mean ± SD (n = 3), including the diameter of the well (9.00 mm).Values with different superscripts in the column differ significantly for the variety and the extraction solvent.

Inhibition activity of extracts was generally higher against *C. albicans* in comparison to the bacterial species. Acetone extracts of the hybrid ginger had the highest activity against *S. aureus* but not different from methanol with the Diameter of Inhibition Zone (DIZ) at 16 mm. This was however lower than the activity of the raw extracts (20.00 mm). There was no significant difference in the antimicrobial activity of ginger extracts against *E. coli*, while *C. albicans* was much more sensitive to the raw extracts (30.67 mm).

Antibacterial activities of the local ginger extracts were generally lower than that of the hybrid. Ethanol and acetone extracts exhibited a non-significant high (13.67 and 13.00 mm) activity against *S. aureus*. DIZ for methanol, ethanol and water extracts *against E. coli* were higher than acetone, though not significantly different. Local ginger was highly effective against *C. albicans* with DIZ ranging from 20.00 to 30.33 mm for methanolic and water extracts respectively. Activity of the water extracts was higher than raw extracts (29.67 mm), though not statistically different (*P > 0.05*) (Table 1).

Garlic extracts were very effective on the three microorganisms tested in the study. The susceptibility of the microorganisms to the different solvent extracts of the two varieties of garlic different significantly (*P* ˂ *0.05*). DIZ of hybrid garlic extracts ranged from 21.33 to 32.67 mm in aqueous and raw extracts against *S. aureus;* 21.33–33.00 mm for methanolic and raw extracts against *E. coli* and 26.00–27.67 mm in ethanolic and water extracts against *C. albicans* (Table 1).

Antibacterial activity of different solvent extracts of local garlic against *S. aureus* did not vary significantly (*P > 0.05*). Water and ethanolic extracts were more effective on *E. coli* (24.00 mm) and *C. albicans* (27.67 mm respectively).

Antimicrobial activity of the raw local garlic was significantly higher than all the solvent extracts for the three microorganisms with DIZ at 29.67, 29.67, and 30.33 mm for *S. aureus*, *E. coli* and *C. albicans,* respectively. Water and ethanolic extracts of local garlic exhibited a significantly high activity against *E. coli* (24.00 mm) and *C. albicans* (27.67 mm) compared to other extracts. Generally, garlic exhibited an effective antimicrobial activity, therefore it was chosen for the MIC study.

### 3.2 Minimum inhibitory concentration (MIC) of garlic in different solvent extracts

The MIC of hybrid garlic of different solvent extracts and the Diameter of the Zone of Inhibition (DIZ) for different concentration of the solvent extracts is summarised in Table 2. Inhibition of extracts against *S. aureus*, *E. coli* and *C. albicans* varied significantly (*P ˂ 0.001*).

**Table 2:** Minimum Inhibitory Concentration (MIC) of solvent extracts of hybrid garlic.

Spice Type			Inhibition Zones (mm)		
	Solvent	Concn mg/mL	*S. aureus*	*E. coli*	*C. albicans*
Hybrid Garlic	Acetone	15	14.33 ± 0.58^de^	14.33 ± 0.58^c^	20.67 ± 0.58^h^
		10	14.44 ± 1.15^de^	14.67 ± 0.58^c^	16.67 ± 0.58^f^
		5.0	13.67 ± 0.58^cd^	13.67 ± 0.58^bc^	17.33 ± 0.58^fg^
		2.5	10.00 ± 0.00^a^	12.67 ± 0.58^b^	15.33 ± 0.58^de^
	Ethanol	15	15.67 ± 0.58^fg^	14.67 ± 0.58^c^	23.67 ± 0.58^j^
		10	15.00 ± 0.58^ef^	13.67 ± 0.58^bc^	21.33 ± 0.58^j^
		5.0	10.00 ± 0.00^a^	10.00 ± 0.00^a^	16.33 ± 0.58^ef^
		2.5	10.00 ± 0.00^a^	10.00 ± 0.00^a^	10.00 ± 0.00^a^
	Methanol	15	13.67 ± 0.58^cd^	14.33 ± 0.58^c^	18.67 ± 0.58^h^
		10	11.33 ± 0.58^b^	13.67 ± 0.58^c^	17.33 ± 0.58^fg^
		5.0	11.33 ± 0.58^b^	10.00 ± 0.00^a^	14.67 ± 1.15^cd^
		2.5	10.00 ± 0.00^a^	10.00 ± 0.00^a^	13.67 ± 0.58^c^
	Water	15	16.33 ± 0.58^g^	16.67 ± 1.15^d^	18.33 ± 0.58^gh^
		10	14.33 ± 0.58^de^	14.00 ± 1.00^c^	15.33 ± 0.58^de^
		5.0	13.00 ± 0.58^c^	13.67 ± 0.58^bc^	12.33 ± 0.58^b^
		2.5	10.00 ± 0.00^a^	10.00 ± 0.00^a^	10.00 ± 0.00^a^
P-value			***0.001***	***0.001***	***0.001***

Values = Mean ± SD (n = 3), including the diameter of the well (9.00 mm). Values with different superscripts in the column differ significantly for the variety and the solvent. DIZ ≤10.00 mm is considered as no activity.

The inhibition effect of hybrid garlic against *S. aureus* started at concentration of 5.0 mg/mL for methanol, water and acetone extracts, while inhibition by ethanol extract (15.00 mm) started at 10 mg/mL.

Inhibition zones of solvent extracts on *E. coli* was observed in concentrations ranging from 2.5 mg in acetone, 5.0 mg/mL in water respectively, while there was no inhibition by ethanol and methanol extracts at the same concentration.

Methanol and acetone extracts significantly suppressed growth of *C. albicans* at concentration of 2.5 mg/mL with DIZ at 13.67 and 15.33 mm. Inhibition activity of water and ethanolic extracts were observed at 5.0 mg/mL (DIZ = 12.33 and16.33 mm, respectively) (Table 2).

### 3.3 Minimum inhibitory concentration of local garlic solvent extracts

The MIC of Local garlic of different solvent extracts is presented on Table 3. The DIZ of the different extracts varied significantly with the concentration of the extracts. All extracts inhibited the growth of *E. coli* and *C. albicans* starting at concentration of 2.5 mg/mL.

**Table 3:** Minimum Inhibitory Concentration of different solvent extracts of Local garlic.

Spice Type			Inhibition Zones (mm)		
	Solvent	Concn mg/mL	*S. aureus*	*E. coli*	*C. albicans*
Local Garlic	Acetone	15	16.00 ± 1.00^fg^	17.67 ± 0.58^jk^	18.67 ± 0.58^c^
		10	15.33 ± 1.53^ef^	16.67 ± 0.58^hij^	17.67 ± 0.58^bc^
		5.0	15.00 ± 1.00^de^	16.00 ± 1.00^def^	16.33 ± 0.58^ab^
		2.5	13.67 ± 0.58^cd^	15.33 ± 0.58^bde^	15.33 ± 0.58^a^
	Ethanol	15	20.00 ± 1.00^i^	18.33 ± 0.58^k^	25.00 ± 1.00e
		10	17.33 ± 0.58^h^	17.33 ± 1.15^ijk^	21.33 ± 1.15^d^
		5.0	14.33 ± 0.58^cd^	14.67 ± 1.15^bc^	18.33 ± 0.58^c^
		2.5	14.67 ± 0.58^cd^	13.67 ± 0.58^ab^	16.33 ± 0.58^ab^
	Methanol	15	15.33 ± 1.15^ef^	15.00 ± 1.00^bcd^	18.67 ± 0.58^c^
		10	13.67 ± 0.58^cd^	15.00 ± 1.00^bcd^	16.33 ± 0.58^ab^
		5.0	10.00 ± 0.00^a^	14.67 ± 1.53^bc^	15.33 ± 0.58^a^
		2.5	10.00 ± 0.00^a^	14.33 ± 1.15^abc^	14.67 ± 0.58^a^
	Water	15	16.33 ± 0.58^gh^	16.33 ± 0.58^eg^	25.67 ± 1.53^e^
		10	14.33 ± 0.58^cd^	15.67 ± 0.58^de^	22.33 ± 1.53^d^
		5.0	13.33 ± 0.58^c^	13.67 ± 0.58^abc^	21.00 ± 2.65^d^
		2.5	11.67 ± 0.58^b^	13.00 ± 0.58^a^	15.67 ± 0.58^a^
***P-value***			***0.001***	***0.021***	***0.001***

Values = Mean ± SD (n = 3), including the diameter of the well (9.00 mm). Values with different superscripts in the column differ significantly for the variety and solvent. DIZ ≤10.00 mm is considered as no activity.

Generally, the inhibition zones increased with the increase in the extract concentration. Higher zones of inhibition for the different extracts at all concentrations were observed against *C. albicans* compared to *S. aureus* and *E. coli.* At concentration of 2.5 mg/mL, *C. albicans* was highly susceptible with DIZ at 14.67 15.33, 15.67 16.33 mm in methanol, acetone, water and ethanol extracts respectively.

Extracts of water, acetone, and ethanol were effective in suppressing growth of *S. aureus* at 2.5 mg/mL and methanol extract at 10 mg/mL respectively. Inhibition effect of acetone extract (15.33 mm) was significantly higher than other extracts at 2.5 mg/mL against *E. coli.*

## 4 Discussion

### 4.1 Effect of solvent extractions on antimicrobial activity of ginger extracts

Hybrid and local ginger exhibited inhibition effect in both aqueous and organic solvent extracts against all the microorganisms tested. This matches previous reports that ginger possesses antimicrobial properties (Abdalla and Abdallah, 2018; Khashan, 2014). Antimicrobial activity could be attributed to the presence of gingerol and shogaol (phenolic compounds) which are active ingredients in ginger (Ali Hasan, 2012). The antimicrobial activity of ginger is reported to depend on the chemical composition, extraction solvent and method of (Beristain-Bauza et al., 2019; Naji and Jassemi, 2010; Park et al., 2008).

The study observed a significant variation in the susceptibility of the three microorganisms tested against the solvent extracts in comparison to the raw ginger juice. Raw, acetone and methanol extracts of hybrid ginger had very high inhibitory at 25 mg/mL, while water and ethanol extracts had slight inhibition effect on *S.* a*ureus.* This is in line with previous findings; methanolic extracts (16 mm) at 25 mg/ml (Ali Hasan, 2012), water and ethanol (9 and 13 mm) at 20 mg/mL (Akintobi et al., 2013).

In other studies, ethanol and methanol extracts of ginger were effective on both *E. coli* and *S. aureus* at higher concentration (Beristain-Bauza et al., 2019). Effectiveness of extracts against *S. aureus* is of great importance in food application as Staphylococcal food borne illness is a serious public health challenge (Kadariya et al., 2014).

It was observed that ethanolic extracts of ginger had antimicrobial effect against some multidrug resistant human pathogens, with the efficacy increasing with concentration (Karuppiah and Rajaram, 2012). However, the antimicrobial activity of the heated extracts was lower than that of the non-heated extracts. This justifies the use of fresh ginger rhizomes and cold extraction for antimicrobial uses as heat destroys the bioactive compounds in the extracts.

In comparison to the organic solvent extracts, inhibition effect of the aqueous and raw extracts of local ginger was very high against *C. albicans*. This is in agreement with findings, where high inhibition by aqueous compared to ethanolic extract against the *C. albicans*, and *E. coli* was reported (Adetunde et al., 2014).

However, these findings need to be investigated further as extraction of spices using water is safe, cheap and could be of interest for industrial food application. On the contrary, other than variation resulting from the extraction method and extraction solvent, studies on the diversity of ginger reported that there is variation in rhizomes and high influence of environmental factors on content of key compounds (Kizhakkayil and Sasikumar, 2011).

This study observed low inhibitory effect of aqueous and organic solvent extracts of fresh ginger against *E. coli*. Whereas other studies have reported no inhibitory effect of aqueous and ethanolic extracts (Akintobi et al., 2013); methanolic extracts (25 mg/ml) of fresh ginger (Ali Hasan, 2012). This could confirm reports that Gram negative microorganisms are more resistant to the ginger extracts than Gram positive microorganisms.

Anti-fungal activities of all extracts were higher than antibacterial activity in both hybrid and local varieties. This is consistent with previous reports that fungi are more sensitive to compounds in ginger than bacteria (Beristain-Bauza et al., 2019).

In another study, MIC values of ginger oil against *C. albicans* and *Aspergillus niger* were much lower than those for bacteria in the same study (Sharma et al., 2013). The high inhibitory effect observed against *C. albicans* could be attributed to the presence of monoterpene, which is reported to have a wide range of antifungal activity (Ali Hasan, 2012).

### 4.2 Effect of different solvent extractions on antimicrobial inhibition activity of garlic extracts

This study confirmed that garlic possesses a strong antimicrobial potential. Aqueous and organic solvent extracts of hybrid and local garlic exhibited high antimicrobial activity against all the tested micro-organism at a concentration of 25 mg/mL. This is in agreement with previous reports in which garlic exhibited strong inhibitory effect against 20 different serotypes of *E. coli*, including the enterohemorragic and enterotoxigenic *E. coli* (Indu et al., 2006). This finding could be of great interest to the food industry as *E. coli* is a major food borne pathogen.

Organic solvent extracts of the hybrid variety were more effective on *S. aureus* compared to the aqueous extracts. Similar findings were reported when white and purple skin garlic cultivars delivered in organic solvents were compared to water-based emulsions (El-Sayed et al., 2017). These results could inform the choice of extracts to be used in food application. On the other hand, aqueous extracts produced the best inhibition activity against *E. coli*. In a previous study, water extraction of garlic at low pH produced the best antimicrobial activity compared to ethanol and methanol (Chen et al., 2018). Antimicrobial activity of garlic is due to its ability to destroy the structural integrity of the cell membrane which is easily achieved at low pH. Moreover, cell-wall structure differentiating Gram-positive from Gram-negative species was previously suggested to influence effectiveness of plant extract (Michielin et al., 2009).

Generally, inhibition effect of the raw garlic was much higher than aqueous and organic solvent extracts of the hybrid and local variety against *S. aureus, E. coli and C. albicans.* This may be attributed to the nature of allicin (the active ingredient in garlic) which is reported to be unstable during processing of garlic because of exposure to varying temperatures, pH, light and extraction medium (Wang et al., 2015).

However, inhibition effect on *C. albicans* was much higher than that of bacteria for the extracts in the two varieties. This is in line with previous studies that investigated antifungal activity of garlic against *C. albicans* (Suleiman and Abdallah, 2014). High inhibition to fungi is attributed to the activity of allicin which is known to curb the performance of some enzymes that is important to fungal growth and activity (Muhsin et al., 2001).

### 4.3 Minimum inhibitory concentration (MIC) of garlic extracts

MIC is the lowest concentration of an antimicrobial agent that inhibits microbial growth in an appropriate medium after incubation (Mostafa et al., 2018). Antimicrobial effects of extracts depended on the concentration; and based on the high inhibition effect of aqueous and organic solvents extracts of garlic at 25 mg/mL, it was appropriate to determine the minimum inhibitory concentration against *E. coli*, *S. aureus* and *C. albicans.*

MIC varied significantly among extracts against the tested microorganisms ranging from 2.5 to 10 mg/mL. Aqueous, acetone and ethanol extracts of local garlic was effective in suppressing growth of *S. aureus* at 2.5 mg/mL. At the same concentration, all extracts of local garlic inhibited growth of *E. coli* and *C. albicans*. Contrary to these, hybrid garlic extracts did not inhibit growth of *S*. *aureus* at 2.5 mg/mL.

In other studies, MIC of 2 mg/mL was reported for *S. aureus* and *E. coli;* while a range of 0.5–2 and 1–5 mg/mL was reported for Gram positive and Gram negative bacteria respectively (Kim et al., 2002). MIC of 0.5–32 and 8.0–64 mg/mL was reported for extracts of white and purple garlic against different strains of streptococci (Groppo et al., 2007); while 5 mg was reported for fresh garlic extract on *E. coli* isolates (Vishal Gaekwad, 2013).

All extract of local garlic and hybrid garlic extracts of methanol and acetone significantly suppressed growth of *C. albicans* at 2.5 mg/mL. This is similar to previous reports in which MIC of 2.5 mg/mL was reported for crude garlic powder against a range of fungi (Suleiman and Abdallah, 2014).

Deviations in MIC are attributed to the chemical variation within the different extracts and the concentration of bioactive compounds within extracts due to the nature of the solvents. For instance, variation in chemical characteristics and antimicrobial activity was noted when 3 different cultivars of Australian grown garlic were compared (Phan et al., 2019). Moreover, Chinese garlic was reported to have high potential as an ingredient in food supplement products due to its high alliin content (Sommano et al., 2016).

It is confirmed that allicin which is the main antimicrobial component of garlic vary in concentration between varieties and extraction/processing and handling of garlic (Cutler and Wilson, 2004; Prati et al., 2014; Shobana et al., 2009). Sensitivity of the microorganisms to aqueous and organic garlic solvent extracts increased with concentration. This trend was observed by other researchers (Indu et al., 2006; Vishal Gaekwad, 2013).

Less active, moderately active and highly active were reported for 10–20, 40–60 and 80–100 mg/mL of garlic extracts on *S. aureus* respectively (Khashan, 2014). Increasing the concentration of the extract also increases the concentration of the antimicrobial compounds in the extracts; hence, positive influence on microbial sensitivity.

## 5 Conclusion

Our study indicates that ginger and garlic (local and hybrid) varieties possess antimicrobial activities against *S. aureus*, *E. coli* and *C. albicans.* Anti-fungal activities of the aqueous and organic solvent extracts were higher than the antibacterial activities. Solvent extracts varied in their antimicrobial effect; raw extract juices were more effective than the aqueous and organic solvent extracts. Generally, garlic exhibited higher antimicrobial activity compared to ginger; although ginger was more potent as an antifungal agent. MIC of garlic extracts varied from 2.5 to 10 mg/mL and the inhibition effect was concentration dependent. Therefore, ginger and garlic consumed in Uganda have promising antimicrobial compounds which can be used in medicine and food application. However, further research is required to document the bioactive compounds in the spices to enhance its application.

## Declarations

### Author contribution statement

Jolly Oder Akullo, Beatrice Kiage, Dorothy Nakimbugwe: Conceived and designed the experiments; Performed the experiments; Analyzed and interpreted the data; Wrote the paper.

John Kinyuru: Conceived and designed the experiments; Performed the experiments; Analyzed and interpreted the data; Contributed reagents, materials, analysis tools or data; Wrote the paper.

### Funding statement

This work was supported by German Academic Exchange services (10.13039/501100001655DAAD)/Regional Universities Forum for Capacity Building in Agriculture (RUFORUM), In-region, In-country Doctoral Scholarship programme 2018 (91712043).

### Data availability statement

Data will be made available on request.

### Declaration of interests statement

The authors declare no conflict of interest.

### Additional information

No additional information is available for this paper.

## Acknowledgement

The authors appreciate the access to research facilities and technical and general support provided by Jomo Kenyatta University of Agriculture and Technology-Kenya and Busitema University -Uganda.
